# Supplementary material for: Exact conditions for evolutionary stability in indirect reciprocity under noise
Source: PLoS Comput Biol. 2025 Oct 14;21(10):e1013584. doi: 10.1371/journal.pcbi.1013584 (PMC12558610; doi:10.1371/journal.pcbi.1013584)
Supplement: S1 Appendix — For each case, we present the norms that satisfy the CESS criteria and the corresponding parameter regimes. (PDF) [file pcbi.1013584.s001.pdf]

Appendix: Exact conditions for evolutionary stability in indirect reciprocity under noise

Nikoleta E. Glynatsi[1,2*], Christian Hilbe[3],Yohsuke Murase[1,2,4]

1 RIKEN Center for Interdisciplinary Theoretical and Mathematical Science (iTHEMS), Wako, Japan
2 RIKEN Center for Computational Science, Kobe, Japan
3 Interdisciplinary Transformation University, Linz, Austria
4 Graduate School of Science and Engineering, Saitama University, Saitama Japan

* nikoleta.glynatsi@riken.jp

1 Numerical verification of ESS norms

To explore whether a social norm is stable, we explore whether players have an incentive to deviate. This in turn depends on the reputational consequences of a deviation. We consider a resident population with action rule $S(X,Y)$ and assessment rule $R(X,Y,A)$. Suppose now there is also an infinitesimal number of mutant players with action rule $S'(X,Y)$. We do not study deviations in the assessment rule because rare mutants have no influence on how the population assigns reputations, see Ref. [**?**]. Let $H(t)$ denote the fraction of mutants with good reputation. When the resident population is at the steady state, $H(t)$ evolves as follows,

$$\begin{aligned}\dot{H}(t) = {} & h^* H(t) R_{S'}(G,G) \\ & + h^*\big(1-H(t)\big)R_{S'}(B,G) \\ & + (1-h^*)H(t)R_{S'}(G,B) \\ & + (1-h^*)\big(1-H(t)\big)R_{S'}(B,B) \\ & - H(t).\end{aligned} \tag{1}$$

In the above equation, we used the following notation for the expected reputation of the mutant donor,

$$R_{S'}(X,Y) \equiv \widetilde{R}\left(X,Y,S'(X,Y)\right). \tag{2}$$

We do not need to take into account cases in which a mutant meets another mutant because mutants are infinitesimally rare. After a sufficiently long time, $H(t)$ converges to the unique stable fixed point

$$H^* = \frac{h^* R_{S'}(B,G) + (1-h^*)\, R_{S'}(B,B)}{1 - h^* R_{S'}(G,G) + h^* R_{S'}(B,G) - (1-h^*)R_{S'}(G,B) + (1-h^*)R_{S'}(B,B)}. \tag{3}$$

Using these stationary values, the probability that a mutant takes A against a resident is

$$\begin{aligned}p_A^{\text{mut}\to\text{res}} = {} & H^* h^* \chi'_A(G,G) + H^*(1-h^*)\chi'_A(G,B) \\ & + (1-H^*)h^*\chi'_A(B,G) + (1-H^*)(1-h^*)\chi'_A(B,B),\end{aligned} \tag{4}$$

where we defined $\chi'_A(X,Y)$ analogously to $\chi_A(X,Y)$ as follows,

$$\chi'_A(X,Y) \equiv \begin{cases} 1 & \text{if } S'(X,Y) = A \\ 0 & \text{otherwise.} \end{cases} \tag{5}$$

Conversely, the probability that a resident takes A against a mutant is

$$p_A^{\text{res}\to\text{mut}} = H^*h^*\chi_A(G,G) + H^*(1-h^*)\chi_A(B,G) + (1-H^*)h^*\chi_A(G,B) + (1-H^*)(1-h^*)\chi_A(B,B). \tag{6}$$

Therefore, the payoffs of the resident and the mutant are

$$\begin{cases}\pi_{\text{res}} &= (b-c)\, p_C^{\text{res}\to\text{res}} \\ \pi_{\text{mut}} &= b\, p_C^{\text{res}\to\text{mut}} - c\, p_C^{\text{mut}\to\text{res}}.\end{cases} \tag{7}$$

The resident is strictly stable against the mutant when $\pi_{\text{res}} > \pi_{\text{mut}}$. A social norm is an ESS when the resident is strictly stable against all possible mutants with different action rules. There are $2^4 - 1 = 15$ possible action rules to check in order to determine whether a social norm is an ESS.

The same analysis can be done for the model with costly punishment. In this case, the payoffs of the resident and the mutant are

$$\begin{cases}\pi_{\text{res}} &= (b-c)\, p_C^{\text{res}\to\text{res}} - (\alpha+\beta)\; p_P^{\text{res}\to\text{res}} \\ \pi_{\text{mut}} &= b\, p_C^{\text{res}\to\text{mut}} - c\, p_C^{\text{mut}\to\text{res}} - \beta\, p_P^{\text{res}\to\text{mut}} - \alpha\, p_P^{\text{mut}\to\text{res}}.\end{cases} \tag{8}$$

The number of different action rules to check in this case is $3^4 - 1 = 80$.

2 Leading norms with punishment action

From the conditions for a CESS norm, we comprehensively derive the leading norms with deterministic rules. Since $S(G,B)$ must be either D or P while $S(B,G)$ may be arbitrary, there are $2 \times 3 = 6$ cases to consider. Note that $R(B,B,C)$ and $R(B,B,D)$ are arbitrary. The optimal action $S(B,B)$ depends on those entries and the game parameters $\{b, c, \alpha, \beta\}$. These six classes of norms are shown in Table 1.

The norms in the first class correspond to the leading eight norms. The players maintain cooperation by cooperating with good players, i.e., $S(G,G) = C$ and $R(G,G,C) = 1$. When a player does not cooperate, the player is regarded as B ($R(G,G,D) = 0$) and is defected against ($S(G,B) = D$) in the next round as a recipient. A B-player can recover its reputation by cooperating with a good player, $S(B,G) = C$ and $R(B,G,C) = 1$. A donor who defects against a B-recipient maintains a good reputation, $R(G,B,D) = 1$; such a defection is deemed as justified. These rules recover the common behaviors of the leading eight. Here, the P action is not advantageous in any context and it is not used by residents.

The second class is overall the same as the first class. However, instead of defecting against a bad recipient, a good donor now punishes such a recipient, $S(G,B) = P$. Due to the punishment, a B-player suffers a larger payoff loss compared to norms in the first class. Therefore, players are stronger incentivized to cooperate under these norms, and hence cooperation is more easily maintained with a smaller benefit-cost ratio b/c. Punishment of a B-player is justified as the donor maintains a good reputation, $R(G,B,P) = 1$. The norms in this class can maintain cooperation unless the cost for punishment α is too large.

The third class corresponds to the secondary sixteen norms. This class is more permissive than the first class. Here, a B-donor does not cooperate with a G-recipient, $S(B,G) = D$. Nevertheless, the donor recovers its reputation, $R(B,G,D) = 1$. Since these norms are more permissive, they require a higher benefit-to-cost ratio, $b > 2c$, to maintain cooperation.

(Donor rep, Recipient rep) (X, Y)	*Action rule* $S(X, Y)$	*Reputation update based on action* C	D	P	*condition*
(G, G)	C	**1**	0	0 (or 1)$^{\triangleleft}$	$b > c$
(G, B)	D	$*$	**1**	$*$	
(B, G)	C	**1**	0	0 (or 1)$^{\triangleleft}$	
(G, G)	C	**1**	0	0 (or 1)$^{\triangleleft}$	$b + \beta > \max\{c, \alpha\}$
(G, B)	P	0 (or 1)$^{\triangleright}$	0	**1**	
(B, G)	C	**1**	0	0 (or 1)$^{\triangleleft}$	
(G, G)	C	**1**	0	0 (or 1)$^{\triangleleft}$	$b > 2c$
(G, B)	D	$*$	**1**	$*$	
(B, G)	D	$*$	**1**	$*$	
(G, G)	C	**1**	0	0 (or 1)$^{\triangleleft}$	$b + \beta > \max\{2c, c + \alpha\}$
(G, B)	P	0 (or 1)$^{\triangleright}$	0	**1**	
(B, G)	D	$*$	**1**	$*$	
(G, G)	C	**1**	0	0 (or 1)$^{\triangleleft}$	$b > 2c - \alpha$
(G, B)	D	$*$	**1**	$*$	
(B, G)	P	0 (or 1)$^{\triangleright}$	0	**1**	
(G, G)	C	**1**	0	0 (or 1)$^{\triangleleft}$	$b + \beta > \max\{2c - \alpha, c\}$
(G, B)	P	0 (or 1)$^{\triangleright}$	0	**1**	
(B, G)	P	0 (or 1)$^{\triangleright}$	0	**1**	

Table 1. Deterministic CESS norms with punishment. The norms are classified into six cases according to the action rules $S(G, B)$ and $S(B, G)$. The left most column shows the reputations that the donor and the recipient originally have. In the second column, the action rules are shown. The third to fifth columns show the assessment rules. They indicate the probability of being assessed as G when a donor of reputation X meets a recipient of reputation Y and takes action A. In the rightmost column, the conditions for the norm to be a CESS are shown. The symbol $*$ indicates that the value can be either 0 or 1. The symbol $\triangleright$ indicates that the value may be either 0 or 1 when $c > \alpha$; otherwise, it must be 0. The symbol $\triangleleft$ indicates that the value may be either 0 or 1 when $c < \alpha$; otherwise, it must be 0. The assessment rules for the context (B, B) are not shown as these may be arbitrary. Depending on these entries $R(B, B, *)$ and the game parameters $\{b, c, \alpha, \beta\}$, the optimal action $S(B, B)$ is uniquely determined.

The fourth class is the same as the third class except that the donor punishes a bad recipient, $S(G, B) = P$, instead of defecting. As seen in the second class, by introducing punishment, cooperation is stabilized even for a smaller b/c, compared to the third class.

The fifth class is similar to the first class but the way to recover the reputation is different. In the fifth class, P is used by B-donors towards G-recipients, $S(B, G) = P$. Donors who use P become regarded as good, $R(B, G, P) = 1$. Therefore, P may be regarded as an apology rather than a punishment. The apology action P requires a cost α. The greater the cost α, the easier it is to maintain cooperation.

Lastly, the sixth class is the same as the fifth class except that the donor punishes a bad recipient, $S(G, B) = P$, instead of defecting. So, in this class, P is used both for apology and punishment.

As indicated in the Table, a G reputation is always assigned when the donor follows the prescribed action rule. When the donor deviates from the action rule, the reputation may be either G or B depending on the context and the action taken by the donor.
