# Supplementary material for: Exact conditions for evolutionary stability in indirect reciprocity under noise
Source: PLoS Comput Biol. 2025 Oct 14;21(10):e1013584. doi: 10.1371/journal.pcbi.1013584 (PMC12558610; doi:10.1371/journal.pcbi.1013584)
Supplement: Attachment — Submitted filename: Response_letter.pdf [file pcbi.1013584.s003.pdf]

We thank the editor for handling our submission, and we are deeply grateful to the reviewers for their careful reading of our manuscript and their constructive feedback. Their comments have greatly improved both the presentation of our results and the overall readability of the paper.

In this document, we provide detailed replies to each comment. In addition, we summarize the main revisions to the manuscript as follows:

- We now emphasize more clearly in the introduction the novelty of our work and how it advances the field beyond previous studies.
- The introduction has been revised to better outline the structure of the paper and to provide a roadmap of the results.
- We have clarified definitions of key terms (e.g., deterministic vs. stochastic norms, assessment errors, ALLD) to improve accessibility for a broader readership.

Reviewer #1: Summary of the paper

This paper provides an analytical method to determine if a given social norm, under indirect reciprocity, can theoretically sustain human cooperation in the donation game. In addition, it extends this analysis to commonly studied scenarios, such as non-vanishing error rates, and the presence of punishments. It uses these criteria to rediscover the leading eight and secondary social norms, providing a formalized understanding of why these social norms are successful.

General appreciation

The paper is written with attention and is structured in an adequate manner. It is clearly motivated as a generalised model of Murase et al., with well-justified improvements that are certainly helpful for future works of indirect reciprocity and human cooperation. While this work is largely a build up from prior works, it makes meaningful contributions and provides enough material to make it a stand on its own. In particular, prior works typically relied on simulations, or analytical approaches with simplifying assumptions which this work does not rely on. As such, it constitutes a technical advance in the field.

Reply: We sincerely appreciate the reviewer's positive assessment of our work and their encouraging remarks on its contributions.

Major issues

Your work assumes a single strategy is present in the population, potentially invaded by a second strategy. Yet, other works in indirect reciprocity consider the potential simultaneous presence of 3 strategies: Always cooperate, always defect, and DISC. Is your analysis capable of expressing such a 3-strategy system? And how does the predicted success of each norm following your conditions differ when these 3 strategies are at play? If differences exist, they should be clarified in the discussion, or explored in an appendix. This would greatly aid the field, as many results of indirect reciprocity are incompatible and often contradictory due to their differing assumptions.

Reply: Thank you for this helpful feedback. The reviewer is correct that our analysis assumes a fixed assessment rule with a single resident action rule, while testing whether a rare mutant with a different action rule can invade. This follows directly from the definition of an ESS: stability is determined by whether any rare mutant can obtain a higher payoff than the resident. So at most two action rules need to be considered at a time, with the mutant forming only a small fraction of the population. We do not study the simultaneous coexistence of multiple action rules, which is an interesting direction for future work but beyond the scope of this paper.

Changes: We added an explicit explanation of why our analysis focuses on at most two action rules at a time, linking it to the ESS framework (Lines 125–133). We also added a paragraph discussing future directions for research on polymorphic populations. (See Discussion)

Following the point above, in Line 336 it would be a helpful addition to clarify what then differs in the evolutionary simulations and your work. That is, why is L6 commonly reported as the most successful norm? Is it a case of misreporting, or are the experiments developed in other works using different settings?

Reply: We thank the reviewer for this insightful comment. We provide intuition in comparison to L3 (lines 389-393, Fig. 3), where we show that L6 exhibits a larger payoff difference between residents and mutants. This difference may explain why L6 is more resistant to invasion, even though the critical b/c ratio is the same for L3 and L6.

Changes: We revised our manuscript so that the above point becomes clearer. (See 4.3)

In Line 398, the connection to reinforcement learning is clear, yet under explored. Is there any insight your methods could bring to reinforcement learning, given that many new works are attempting to implement reputations to promote cooperation in multi agent systems?

Reply: We thank the reviewer for highlighting this connection. Our main contribution is to illustrate the long-term benefit of maintaining a good reputation and to examine whether changes in the action rule can increase payoffs. In this sense, our approach is loosely related to reinforcement learning, as strategy adjustments can be viewed as responses to payoff changes.

That said, we do not claim that our framework directly translates to multi-agent systems. Since we consider a large population where a mutant's behavior does not affect the residents' payoff, our method has a link to single-agent settings rather than multi-agent ones. Furthermore, our method hinges on the binary reputation assumption. For these reasons, we feel that our method will not be easily applicable to multi-agent reinforcement learning.

Minor issues

Line 28: Clarifying what a deterministic or a stochastic norm is could aid the reader.

Line 52: Without reading ahead, it is unclear what a secondary norm is.

Line 204: Although it is clear for readers familiar with indirect reciprocity, ALLD should be defined, especially S(X,Y), since otherwise it is unclear why Eq 19 is satisfied.

Reply: We thank the reviewer for these helpful suggestions. We agree that clarifying these terms improves accessibility.

Changes: To address these comments, we now defined deterministic and stochastic norms in the Introduction (Lines 28–29 and 34–35); we defined the order of norms in the Model section (Lines 84–93); and we defined ALLD explicitly in Line 234.

Line 65: While I understand the usage of "build-up a reputation over time", the model then explicitly states that reputations are binary and players only consider the current reputations, which may be confusing to the reader.

Reply and changes: That is right; we have rephrased the sentence to avoid this kind of confusion. Instead, we now say that their actions can affect their reputation, which in turn may influence how they are treated in future.

The author summary states that social norms define how reputations change, but, from the remaining text, social norms appear to only have this effect indirectly, as they instead define what is considered good. It is better to clarify this.

Reply: We thank the reviewer for this helpful observation. We agree the phrasing could be misleading. Social norms specify how actions are judged, which in turn determines how reputations are updated.

Changes: We now write: “shared rules that specify how actions are judged and thereby how reputations are updated.”

Line 179: There’s a small inconsistency, it should read “second-order”.

Line 379: Small typo at “[…] enables us to study of a wider […]”

Reply and Changes: We have corrected both typos. Thank you for spotting them.

Equation 7: It is better to clarify the superscript “res->res” for the reader, as only in the appendix is a different usage of the notation present.

Reply and Changes: We have clarified the meaning of the superscript and introduced the notion of the resident norm in the main text. Specifically, in Section 3.1, we added a sentence introducing the resident norm, and we added a sentence explaining that “res” denotes a resident individual.

Line 306: From the punctuation and phrasing, the two conditions are hard to understand. In my first reading, I understood that a social norm is a CESS if either S(B, G) = C, or it satisfies Eq. 41, which itself also contains S(B, G) = C, and only in line 309 did the other condition make sense.

Reply and Changes: We thank the reviewer for pointing this out. We agree the phrasing was confusing and have revised the text to make the two conditions clearer (see Lines 343–356).

Reviewer #2: I want to congratulate the authors to their very comprehensive work. I love to see the field of indirect reciprocity continue to evolve. I also want to state, that I am familiar with the topic of indirect reciprocity, yet that I do not have a lot personal experience with the analytical approach they apply. I tried to understand the methodology as good as possible, but I cannot make a definitive statement on its soundness.

Reply: We thank the reviewer for their careful and thoughtful reading of our manuscript, as well as for their encouraging remarks about its contributions. We also appreciate the critical questions raised about structure, scope, and accessibility.

The authors work seems to be mainly based on their own previous studies [21] and [23], which tackled costly punishment (and various errors), and stochastic third-order norms respectively. In the current study, these aspects seem to be combined in one model for the first time. The authors use their model to study 4 specific cases:

1) What strategies are CESS if there are no errors – the result is a list of conditions for two classes of stochastic strategies. The subset of these which are deterministic instead reproduces the leading-eight and the secondary-sixteen norms from previous studies. This is a validation of their and the previous work.
2) Which are CESS if costly punishment is an option (apparently also if there are no errors) – the result is another list of conditions and a list of classes of deterministic third-order strategies, the second-order subset again reproducing previous work.
3) Under which condition are the leading-eight CESS under diverse errors (3 types) – The results are conditions, as well as figures that show the values of the errors for which the model and a numerical simulation show each leading-eight to be ESS. Some finer points are made about L3 and L6.
4) Description of equalizer norms (apparently again if there are only assessment errors) – This type of norm provides the same payoffs for cooperation and defection. As a result, mutants cannot earn higher payoffs than

residence. The general principle is described, but conditions are only given for GSCO (another reproduced finding) and another example for which the central equation could be simplified.

In the discussion, the authors also state that their model has advantages over previous ones, the difference being the use of "Delta v" = the difference between the payoffs for a player with good reputation compared to one with bad reputation. The authors contrast this with the approach of [13].

I consider the successful reproduction of previous works is an important contribution to the field. The paper might be the first to test the stability of the leading-eight under public assessments with these specific types of errors or three different types of errors simultaneously in general. The discovery of additional CESS that use costly punishment and of other 'zero-determinant'-like strategies are important findings as well. However, I would like to ask the authors to address some concerns about the structure of the paper, the accessibility to less familiar reader and some other points. Especially in the beginning, but also later one, I asked myself some broader questions: Is this paper foremost about the model, or about the results? Why do you introduce various errors to then not use them in 3 out of 4 cases? (without commenting on it) OR Why were all model extension combined in a single paper, but then only applied one at a time?

Reply: We thank the reviewer for their careful and thoughtful reading of our manuscript, as well as for their encouraging remarks about the contributions of our work. We wish to note that the reviewer may have overlooked one class of results, namely our characterization of ESS at any cooperation level in the presence of errors.

We also appreciate the critical questions raised about the structure and focus of the paper. Our main finding in this paper is the theorem in Section 3.3. The theorem is also useful for the analysis of ESS under various errors (Section 3.4), and it is straightforward to extend the analysis to the case with additional actions (Section 3.5). Although these extensions can be taken into account simultaneously, we present each one at a time in order to clarify the consequences of each extension.

We think this misunderstanding arose from the way we originally presented our results. To address this, we have now improved the introduction to better outline the overall contributions, and clarified the structure of the paper.

Changes: We revised the Introduction section such that our main findings and contributions are more clearly articulated.

I will list points I think addressing could potentially improve the paper, and I will mark some of them as [MAJOR] that would be more essential.

General issues:

The scope of the paper is quite large. The actual result sections, on the other hand, seem rather short. Some results are not even properly shown or discussed in their section (see some detailed comments below). Which makes the paper hard to grasp. It is almost as if a clear story is missing. A solution could be to split it up the paper (a rather drastic move) or move some entire section to the supplements. But it might also benefit from some simple restructuring (e.g. combine the analysis of each case with its results and a short discussion of each in a section; or to keep the topics in the analyses and cases in the same order).

Reply: We thank the reviewer for this valuable suggestion, which aligns with comments from other reviewers. We agree that a clearer structure helps to present the results more effectively.

Changes: We revised the introduction to highlight contributions and novelty more explicitly and added an introductory paragraph to the Special Cases section to provide a roadmap. We hope that together, these changes improve the flow, and that they make it clearer what each result contributes to the overall story.

[MAJOR] Probably as a consequence of the scope, that had to be packed in a few sentences, I find the abstract potentially a bit miss leading. When I first read it, I was expecting the authors would provide a list of all CESS under (various) noise. Instead, they do report all CESS under no noise, but study various noise only for the leading-eight. Also, the costly punishment and equalizer norms seems to be studied without the various noises.

Reply: The reviewer is correct that in Section 4 (Special Cases), we present two sets of results in the noise-free setting. However, we also characterize ESS under various error rates in earlier sections. For this reason, we feel the abstract properly reflects what we do. Having said that, we do agree that it is important to specify the assumptions made in each set of results. We have revised the introduction where we summarize the results, to make these assumptions explicit.

Changes: The introduction now explicitly summarizes the different sets of results (Lines 56–67).

Equations in general could be elaborated on. It sometimes feels like the reader is required to fully understand meanings, reasons and consequences only by the equations themselves to be able to follow the line of argument. It would help if the text provided more information to the equations and speak for itself, also making the transitions between equations easier to follow. It took me a while to understand and it is possible that I only succeeded because I read some analysis of [21] and [23], which seem easier to follow (but are also smaller in scope).

Reply and Changes: Thank you for those comments. We fully agree, some of the equations would indeed benefit from additional context and explanation. To address this comment, Section 3.2 has been revised to include additional explanations and to provide further context for the presented equations. See also the following revisions.

Some phrases are introduced without any explanation. Many researchers will know them, but some might not, I believe it could help to add some information about some of them e.g. Line 28 – “stochastic norms”, Line 93 – “ergodic”, “short memory effect” Line 182, “perfectly discriminating” Line 183, “ALLD” Line 204 (and it is called a norm, but which norm is it? Given all Deltas are zero, it could be all R=0 or all R=1, right? I think ALLD is most often used to describe the action rule and not the norm), “Bellman equation” line 400

Reply and Changes: Thank you for pointing this out, we fully agree. In the revised version, we now define deterministic and stochastic norms in the Introduction (lines 28–29 and 34–35). We also define ergodicity (lines 113–116). Although we no longer use the terms “short-memory effect” and “perfect discrimination,” we explicitly state what we mean by these concepts (lines 210–211 and 212–214). Finally, we define ALLD explicitly (line 234).

Single Issues:
Over the course of reading, I have asked myself a few questions, that might be helpful indications on what could be unclear to a general audience. I don’t ask the authors to answer them all (except the ones I marked as [MAJOR]), but I wanted to provide them all in case they might be helpful.

How is equ 5 derived?

Reply and Changes: We wrote that the equation is derived from Eq.(2), see the sentence before Eq.(4).

What would be a name for X in equ 8?

Reply and Changes: We explicitly state that \chi is an indicator function, see the sentence before Eq.(8).

How can equ 15 be simplified to 16? … It took me a while to realize that the cost in 15 only occurs if a player with a bad reputation has to act differently to a player with a good reputation.

Reply and Changes: We revised the entire paragraph for clarity.

[MAJOR] Line 311: “Those two …” Does that mean that there are no stable stochastic CESS after all? And the leading-eight are not special cases but the only cases for the first and so on?

Reply and Changes: The leading eight and the secondary sixteen are the only CESS when assessment rules are deterministic. In contrast, in the stochastic case there exists a spectrum of CESS. We have revised the text to make this more clear (Lines 353–356).

Line 331: “which is a root of the quadratic equation.” - What is the consequence of that?

Reply and Changes: The consequence is that the expression becomes analytically tractable, but it is too complex to obtain insights. We made it clear in the revised text, see the explanation before Eq.(49).

What is a good summary of the additional inside in 4.1) compared to [23]?

Reply and Changes: We derive the same results as [23] using a different analytical approach in order to demonstrate the validness of our approach. To clarify this intention, we added a sentence, see the first paragraph in Section 4.

Why is the role of L3 and L6 (and their differences) discussed in such detail, even with an additional figure 4, when Fig 5, the main finding is only in the supplements?

Reply: L3 and L6 are the only second-order social norms among the leading-eight, which have been investigated more thoroughly in the literature.

Moreover, because they are the only second-order norms, the expression of \Delta v for those norms becomes particularly simple, which is useful to obtain some intuition. That's why we focused on these results in the main text, while we additionally showed the figures for the other leading eight norms for completeness.

[MAJOR] Why is fig 4 coming after fig 3?

Reply and Changes: Thanks for catching this, we have changed the order.

[MAJOR] Should it be [21] instead of [23] in line 388?

Reply and Changes: Again, thank you for pointing this out. We have updated the reference.

Why are the results (the CESS strategies) of 4.2) only shown in a table in the supplements?

Reply: We have the CESS condition (44) for the general stochastic norm case in the main text. For the readers' convenience, we additionally included a table for the deterministic case in the SI.

Changes: We have revised Section 4.2 and added a reference to Appendix B.

[MAJOR] Is it not true, that table 3 has strategies that were not in previous studies (line 388)? If so, why is this not discussed?

Reply and Changes: Thank you for raising this issue. Indeed, Table 3 contains new results and we discussed them in Appendix B. Since the results for general stochastic norms are already shown in the main text, we opted to put it in the SI. In order to clarify those points, we added a sentence to guide to Appendix B in the revised main text.

[MAJOR] In the beginning of the discussion, the authors state that “most” other studies required error rates that are vanishingly small (which I take to mean effectively 0). Which seems a bit odd given that even (one of) the most fundamental papers [9] in 2004 already considered two types of errors >0. The statement feels a bit like an attempt to boost the importance of the current paper, a statement that I do not really agree with. If

the authors had a list of important papers that required vanishingly small errors in mind, it would be helpful to cite them here.

Reply: We thank the reviewer for this clarification. They are correct that Ref.[9] and many subsequent numerical studies considered non-vanishing positive error rate. On the other hand, their primary interest is in the behaviors when the errors are sufficiently small. Many studies introduced errors to show that the results are robust to these errors. We rephrased the sentences to avoid misunderstanding.

Changes: We revised the sentences in the first paragraph of the discussion.

[MAJOR] What are the "wider range of social norms, including those that tolerate moderate levels of error." Line 379? (Or why would the norms you can look at with your extension be more able to tolerate errors, and how would you know since you only study those errors for the leading-eight?)

Reply and Changes: We agree the phrasing was unclear. We have revised this part to: "This broader framework enables us to study a wider range of social norms, and their stability under arbitrary levels of error." (Line 426-428).

[MAJOR] What are the 'leading norms' in that context? Line 386

Reply and Changes: Here we mean cooperative ESS. The text has been updated for clarity.

The use of "Delta v" is introduced as an important improvement over past models Line 397. However, with the information given in the discussion, I was not clear why that is the case. It might be my fault for not understanding the details enough. But for the sake of readers like me, the other approaches could be explained in more detail, and what makes this one better.`

Reply and Changes: We have revised the discussion to explain more clearly why Δv is central and how it simplifies the analysis compared to earlier approaches.

At last, maybe also consider the following very minor points:

Table 1 - Ohtsuki and Iwasa [9] characterized L1 as "standing" based on "Sugden, R., 1986. The Economics of Rights, Co-operation and Welfare. Blackwell, Oxford, UK."

Reply and Changes: We have updated Table 1 accordingly.

Line 88 – introduction of errors, which are only later specifically called "assessment errors" (It is in the sentence, but if one searches for the phrase "assessment error" they do not find this passage)

Reply and Changes: We agree, we now introduce the term assessment error explicitly at Line 106.

Line 108 – citation of previous use of CESS might be helpful

Reply: Indeed, we added appropriate references (Line 137).

Line 276 – citation or explanation why they are interchangeable might be helpful as well

Reply: We added a reference (Line 315).

Again, I find the model and the findings excellent and worthwhile work. I hope the points I raised can improve the quality of the paper even more.

Reply: We thank the reviewer again for their thoughtful comments. We feel this feedback was extremely helpful to improve the clarity, accessibility, and framing of the manuscript.

Reviewer #3: Summary The manuscript develops a generalized framework to characterize analytically the evolutionary stability of social norms in indirect reciprocity under different types of errors. It extends previous work by: allowing non-vanishing error rates, incorporating stochastic norms, and accommodating additional actions beyond cooperation and defection. The paper also identifies a class of "equalizer" norms, drawing an analogy to zero-determinant strategies in direct reciprocity. The paper is well-written, the results are novel and interesting, and the mathematical treatment is sound.

Overall evaluation The manuscript makes a substantial theoretical contribution, but to fully convey its impact it needs clearer framing of its novelty, tighter exposition in the introduction, and expanded explanation of the key methodological controbutions. These are primarily presentation and clarity issues; the underlying mathematics appears sound. So, I recommend a minor revision.

Reply: We thank the reviewer for their clear summary of our work, their positive assessment, and their constructive feedback on how to improve the manuscript.

Major comments [1] The paper would benefit from restructuring to make the novelty of the generalized framework explicit. In particular, clarify how and why this framework: (a) enables calculation of quantities that were previously more complex or intractable, (b) supports extensions such as additional actions beyond cooperation and defection, and (c) leads to the identification of equalizer norms. Bringing these points forward early will help the reader appreciate the contribution.

Reply and Changes: We thank the reviewer for this helpful feedback. To address this comment, we have revised the Introduction to describe the novelty of our work more clearly. In addition, we have expanded the Introduction's last paragraph, to provide a clearer outline of the manuscript's structure.

[2] The paragraph at lines 25–37 mostly describes Ref. [23] to set up the extension. Ref. [23] covers stochastic norms (deterministic as a special case), under public assessment, and derives ESS conditions in the limit of vanishing errors. At this point, it would be helpful to state clearly what this paper modifies or extends. The paragraph ends by hinting that the framework from [23] can be used for private assessment, but this manuscript does not address private assessment. I recommend rephrasing to avoid raising expectations that are not met. The following paragraph (lines 38–48) starts with the restriction to vanishing error rates, which has already been mentioned. Framing it as "Moreover" is confusing. Consider merging or reordering these sections to avoid repetition.

Reply and Changes: We thank the reviewer for spotting this issue. We have revised the Introduction to present the historical contributions and the novelty of our work more clearly. We have also removed the extended discussion of Ref. [23] to avoid ambiguity or creating expectations that we do not meet.

[3] The analysis considers variation only in the mutant's action rule, not in its assessment rule. In the main text, this is assumed without explanation, and Appendix A cites Ref. [9], where it appears as a modeling postulate: "We postulate that reputation dynamics d is fixed in the population." While readers with expertise in the field may infer that public assessment implies a unique or external reputation mechanism that the mutant cannot influence (e.g., an external observer or a random broadcaster which very likely is the resident), this is not obvious in the paper. I recommend adding a short explanation in the main text for why only action-rule deviations are analyzed. It would also be helpful and very interesting to clarify whether the presented framework could, in principle, be adapted to include competition of assessment rules.

Reply: Indeed, as is common when studying public assessments, we assume that mutants only change their action rule while keeping the assessment rule fixed. The respective interpretation is that the reputation-updating mechanism is shared and fixed at the population level, so mutants cannot influence it. Under this assumption, it is sufficient to only consider deviations in the mutant's action rule.

We fully agree with the reviewer that it would be very interesting if this technique could be used to compare different assessment rules. However, as of now we would not know how to accomplish this.

Changes: We now clarify in the main text why only action rule deviations are analyzed under the public assessment model (Lines 127-131). In addition, we now mention in the Discussion that future work should tackle the case of deviations in the mutant's assessment rule.

[4] The discussion (line 396) correctly identifies the use of \Delta v as the key methodological innovation. Because of its importance, Section 3.2 would benefit from a more detailed derivation and added intuition. The expression not only contains current payoffs that depend on the probabilities of the actions, but also future payoffs that depend on the reputation updates. For example, lines 156–159 briefly explain the role of future payoffs through reputation updates; expanding this explanation and adding detail and intuition of the derivation would help readers understand the power of the method.

Reply and Changes: We sincerely appreciate this suggestion! In the revised manuscript, we now provide more details and a better intuition for this \Delta v term, to further clarify its meaning and significance.

[5] In the introduction (line 42), “all evolutionarily stable social norms” is acceptable as shorthand, but in the discussion (line 374), “we analytically characterize all evolutionarily stable norms (ESS) of indirect reciprocity” feels overly strong. The analysis is limited to norms up to third order and to public assessment. Rephrasing both statements to specify this scope would be more accurate.

Reply and Changes: We appreciate the reviewer's careful reading. We now write: “In this paper, we focus on indirect reciprocity under public assessment. Within this setting, we analytically characterize all third-order evolutionarily stable norms (ESS)” (Lines 421-422).

Minor comments

The use of “full cooperation” needs to be clarified. Does it mean all players always choose C? In lines 39–40, what does “default state” means in the phrase “full cooperation is assumed to be the default state”?

Reply and Changes: Indeed, "full cooperation" means that all players cooperate. We no longer speak of a "default state", we agree this phrase was not clear.

The statement that the principles of the leading eight norms “seem to be shared by many human communities” (lines 23–24) must be supported with a citation.

Reply and Changes: Indeed, and we provide now references to relevant experimental papers. However, when adding these references, we also realized that the sentence suggests perhaps a broader consensus than is justified, in light of the published literature. We have thus rephrased the sentence to give a more nuanced view on the empirical evidence. Thank you for making us revisit this statement!

Please specify whether “The theory” in line 34 refers to Ref. [23] or to the general theory of indirect reciprocity. With the current wording, it could be either.

Reply and Changes: We agree that our original wording was ambiguous. To address similar comments from a previous referee, we have now removed this sentence from the introduction. We still discuss how public assessment models can serve as a foundation for analyzing private assessment models in the Discussion section.

In line 411, clarify what is meant by “default reputation.” Is this the initial condition of the reputation dynamics?

Reply and Changes: In the previous analysis in [23], we first derived the condition that the fraction of the good players converges to 1. On top of that, we derived the condition that the norm is an ESS, assuming that the population mostly consists of good players. In that setting, we can consider that being bad is a transient state and that players return to the "default" state of good reputation eventually.

Thanks for pointing out this ambiguity. We rephrased the text for clarity.

In Section 4.2, daggers are used to denote additional actions with punishment, but daggers are also used earlier for errors. Using the same symbol for both is visually confusing; consider refining the notation.

Reply: Thank you for spotting this. We now denote errors with a star, and leave daggers to indicate punishment actions.

The term “social norm” is formally defined as the combination of action and assessment rules, but when referring to the “order” of the norm it seems to mean only the assessment rule. A brief clarification of usage or consistency throughout would be helpful.

Reply: Let us explain: With respect to a norm's assessment rule, a first order norm only takes the player's last action into account; a second order norm considers additionally the respective recipient's reputation; and a third order norm also considers the donor's reputation. Because our action rules depend on both the donor's and the the recipient's reputation, we still interpret them as being third-order (even if the action rule only has two arguments).

Changes: We now provide a definition of a social norm's order in Section 2.

Figure 2 lacks an x-axis label; add it for clarity even if the caption implies the content.

Reply: Thank you, this was a mistake on our part. We have now included the x-axis label.

Figure 4 is referenced immediately after Figure 2 and before Figure 3; and Figures 2 and 4 both compare L3 and L6. Consider swapping Figures 3 and 4 to match the order of discussion.

Reply: We have reordered the figures accordingly. Thank you.

Generous Scoring is described as a first-order norm in the text but labeled second-order in the caption of Figure 3. Standardize this.

Reply: This was a typo in the caption, now corrected.

In Table 3, the triangular inequality symbols would be clearer if the terms were consistently ordered (e.g., use c \alpha and c < \alpha rather than flipping sides) so the symbols match the inequalities.

Reply: Thank you, we have updated the caption of Table 3 for consistency.
